# Clinicopathological Profile of Medullary Thyroid Carcinoma—Could We Predict Aggressive Behavior?

**DOI:** 10.3390/biomedicines11010116

**Published:** 2023-01-03

**Authors:** Simona Eliza Giusca, Elena Corina Andriescu, Irina Draga Caruntu, Delia Ciobanu

**Affiliations:** 1 Department of Morpho-Functional Sciences I—Histology, Pathology, “Grigore T. Popa” University of Medicine and Pharmacy, 700115 Iasi, Romania; 2 Department of Pathology, “Sf. Spiridon” Clinic Emergency County Hospital, 700111 Iasi, Romania

**Keywords:** medullary thyroid carcinoma, clinicopathological characteristics, extrathyroidal extension, lymphovascular invasion, lymph node metastasis, grading system

## Abstract

Medullary thyroid carcinoma (MTC) accounts for only 2–5% of all thyroid malignancies. Clinical and pathological characteristics alone may suffice to predict outcomes, but unstable behavior in some cases suggests that other factors may influence a worse course of the disease. This study aims to identify criteria that could predict increased aggressiveness. We analyzed 59 consecutive MTC cases. We focused on the relationships among clinicopathological characteristics, parameters of aggressiveness (extrathyroidal extension, lymphovascular invasion, and lymph node metastasis), and parameters for MTC grading. Statistically significant correlations were found for tumor size, lymphovascular invasion, and lymph node metastasis and tumor focality and lymph node metastasis. Our results showed, in tumors larger than 40 mm, odds ratios (ODs) of 13.695 and 6 for lymphovascular invasion and lymph node metastasis, respectively; in multifocal tumors, we registered an OD of 9.42 for lymph node metastasis. No significant correlation was found for the parameters of the MTC grading system when assessed individually and integrated by reporting low-grade and high-grade risk groups. Although our data indicate that lymphovascular invasion and lymph node metastasis remain significant markers for aggressiveness, studies on larger series of cases are mandatory to detect and validate new factors responsible for the variable course of MTC.

## 1. Introduction

Medullary thyroid carcinoma (MTC) is a malignant epithelial tumor that accounts for only 2–5% of all thyroid malignancies [1]. However, compared with other thyroid carcinomas (TC) with a mild aggressive course, MTC is responsible for a disproportionately high number of deaths [1].

MTC was defined as an individual pathologic type of thyroid carcinoma (TC) in the late 1960s [2] based on the connection between the parafollicular or C-cells present within the thyroid cell population (originating in the neural crests and synthesizing the polypeptide hormone calcitonin), some particular morpho-functional characteristics, and the clinical course of malignant proliferation. These characteristics exist in a distinct morphological substrate in which the amyloid presence is critical and are associated with the presence of serum calcitonin, moderate aggressiveness, and nonresponsiveness to radioiodine treatment [3,4,5,6]. Therefore, it is also known as C-cell carcinoma, parafollicular cell carcinoma, or solid carcinoma with amyloid stroma.

C-cell hyperplasia (CCH), characterized by an increase in C-cell clusters over 50 cells per low-power, has been identified as a precursor condition for MTC, leading to a progressive evolution towards in situ and subsequent microinvasive MTC [1]. However, CCH is a relatively common condition in middle-aged adults with Hashimoto thyroiditis, hypothyroidism, or hypergastrinemic and hypercalcemic status [1]. Thus, basal calcitonin and calcium-stimulated calcitonin levels can be useful for an early diagnosis of MTC, especially when basal calcitonin is softly elevated [7].

Approximately 75–80% of MTCs occur sporadically while the inherited forms of MTC are responsible for the rest of the cases [8,9,10].

Progressions in deciphering the molecular pathways responsible for MTC pathogenesis confirm the role of germline-activating mutations in RET proto-oncogene, which occurs in hereditary MTCs that develop in multiple endocrine neoplasia type 2 (MEN2) syndromes. Furthermore, such progressions identify several somatic mutations in sporadic MTC [1,8,9].

MTC frequently metastasizes, and the tumor spreads both in hematogenous and lymphatic ways. As such, the lymphatic and vascular invasion may be seen at the pushing front of the tumor. Among patients presenting a palpable thyroid nodule, the incidence of clinical cervical lymph node involvement at the time of diagnosis has been reported to be as high as 75% [6,11].

Recently, in order to optimize the prediction of tumor behavior, an international MTC grading system was proposed, extrapolating the grading systems already implemented for pulmonary and gastrointestinal neoplasms with a neuroendocrine origin [12]. This system is based on three parameters: Two of them, the mitotic index and Ki67 proliferative index, assess the proliferative activity of the tumor; the third counts tumor necrosis [12].

Our interest in MTC is justified by facing this pathology, as the “Sf. Spiridon” Clinic Emergency County Hospital of Iasi is an important center for diagnosis and treatment of thyroid pathology for the northeastern region of Romania with an annual addressability of about 450 patients. Moreover, this region is predisposed to thyroid pathology in the context of iodine deficiency and the Chornobyl nuclear disaster. TC studies in our county are infrequent and often focused on PTC as the main form of thyroid tumor [13,14,15,16,17]. However, reported data from our center showed that MTC was identified in 6.74% of TC, which is more than European studies [17,18]. Although clinical and pathologic characteristics alone may suffice to predict the outcome in most patients, the unpredictable behavior of MTC in some cases suggests that other factors may also influence the disease progression and survival.

Within this context, we analyzed the clinicopathological profile of MTC, pointing to cases with increased oncological risk. Our analysis focused on the possible relationships between a set of clinicopathological characteristics and three parameters expressing tumor extension and aggressiveness (namely, extrathyroidal extension, lymphovascular invasion, and lymph node metastasis) in correlation with the new proposed MTC grading system.

## 2. Materials and Methods

### 2.1. Study Group

The retrospective study comprised 59 consecutive cases of MTC investigated in the Pathology Department of the “Sf. Spiridon” Clinic Emergency County Hospital of Iasi for seven years (2010–2016). The research has been approved by the Ethics Committees of the hospital and “Grigore T. Popa” University of Medicine and Pharmacy, Iaşi, pursuant to the ethical standards of Helsinki declaration regarding the patients’ informed consent for the use of their medical information for scientific purposes. Our database included basic demographic data (gender, age), pre- and postoperative calcitonin value, and clinicopathological parameters.

### 2.2. Histological and Immunohistochemical Evaluation

The diagnosis of MTC based on histological assessment was immunohistochemically confirmed by using the following monoclonal antibodies: chromogranin A, synaptophisin, calcitonin, CD56, TTF1, and Ki67 (Table 1). For chromogranin A, synaptophisin, calcitonin, CD56, and TTF1, the immunoreaction was assessed as negative or positive with low (1+), medium (2+), or high (3+) intensity.

All cases were reviewed by three independent pathologists in order to update the histopathological characteristics concluded at the time of the diagnosis (between 2010 and 2016) in accordance with the 2017 World Health Organization (WHO) Classification, Tumor Node Metastasis (TNM) staging and American Joint Committee of Cancer (AJCC) criteria [19,20,21]. We documented the tumor size, histological variant, focality (two or more foci), lymphovascular invasion, extrathyroidal extension (defined as microscopic presence of tumor cells in perithyroidal muscle tissue), resection margins, lymph node metastasis (including the number of positive lymph nodes), tumor stage, associated thyroid pathology, and tumor recurrence.

### 2.3. Grading System

All cases were evaluated by using the international MTC grading system that includes the mitotic index and Ki67 proliferative index in the highest proliferative parts of the tumor and tumor necrosis in the nonscarring areas [12]. The mitotic index was calculated per 2 mm^2^, meaning 10 high-powered fields [12,22], whereas for Ki67, we counted between 500–2000 tumor cells [12]. Tumor necrosis was labeled as present or absent, taking into consideration tumor cells with degenerating cytoplasm and dots of karyorrhectic nuclear detritus regardless of lesion size [12,22].

The MTC grade was established as low and high, the high-grade tumor requiring at least one of the following three criteria: mitotic index ≥ 5 per 2 mm^2^, Ki67 proliferative index ≥ 5%, and/or tumor necrosis [12].

### 2.4. Statistical Analysis

Statistical analyses were performed using the SPSS V.22-SPSS Inc., IBM Corporation, Chicago, IL, USA. The correlation between the clinicopathological characteristics and aggressiveness parameters, including grading system criteria was assessed using Chi-squared test for categorical variables and Kruskal–Wallis test for continuous variables. The risk of aggressive behavior (odds ratio—OR) in relation to the aggressiveness parameters was assessed using univariate analysis. *p*-value < 0.05 was considered statistically significant.

## 3. Results

### 3.1. General Clinicopathological Characteristics

Among the 59 patients, 55 cases were sporadic MTC and four cases were hereditary MTC with familial tumor aggregation. The classification of the four cases into the familial form was carried out based on patient clinical data. These four patients had a family aggregation between the fourth and fifth degree (cousins and nephews). No genetic tests were performed to evaluate specific mutations.

The small number of familial MTC cases did not allow for a comparative assessment between this category and the sporadic ones. Therefore, we only reported the number of cases belonging to this particular category for each considered clinicopathological characteristic.

In the study group, 46 patients (77.9%) were female, and 13 patients (22.1%) were male; each gender category comprised two cases of familial MTC. The mean age was 59.75 ± 11.12 years; 42 patients (71.19%) were over 55 years old (yo) at the time of diagnosis whereas 17 patients, including those with familial MTC, were under 55 yo (28.81%).

In all cases, surgical treatment consisted of a total thyroidectomy with lymph node dissection in 43 cases (72.88%). Lymph node dissection was not performed in 16 cases (27.12%) because the imaging investigation did not reveal the presence of lymphadenopathy.

The mean tumor size was 27.64 mm ± 2.50 mm and ranged between 2 and 80 mm.

**Figure 1:**
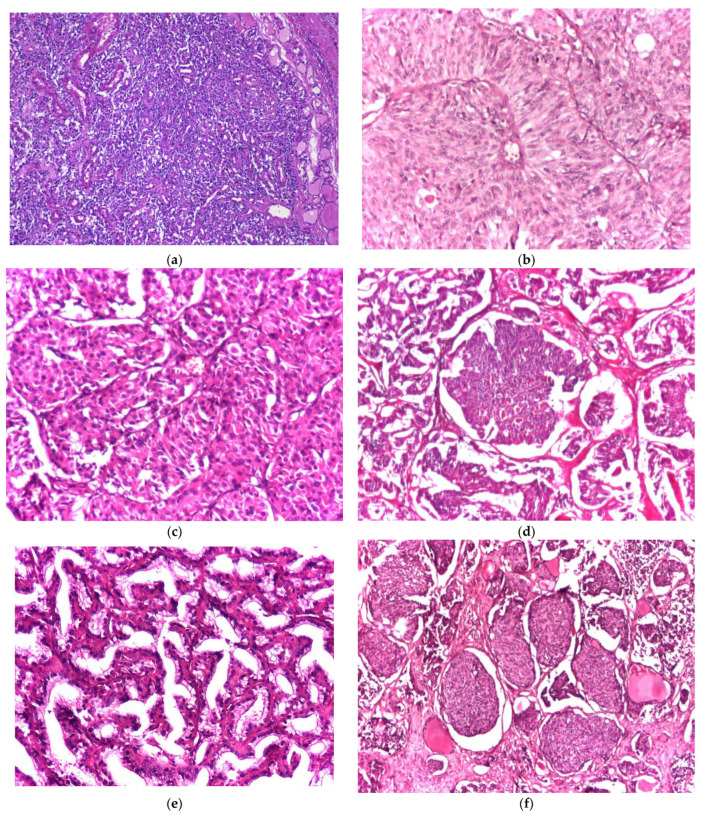
(**a**–**e**) Histological variants of medullary thyroid carcinoma: (**a**) conventional variant (hematoxylin and eosin—H and E, magnification 4×), (**b**) spindle cell variant (hematoxylin and eosin—H and E, magnification 20×), (**c**) oncocytic variant (hematoxylin and eosin—H and E, magnification 20×), (**d**) follicular/glandular variant (hematoxylin and eosin—H and E, magnification 4×), (**e**) pseudopapillary variant (hematoxylin and eosin—H and E, magnification 10×), and (**f**) small cell variant (hematoxylin and eosin—H and E, magnification 4×).

The histopathological exam identified the following variants of MTC: 35 cases (59.32%) with four of them being familial MTC—conventional, 12 cases (20.33%)—spindle cell, five cases (8.47%)—oncocytic, two cases (3.38%)—pseudopapillary, three cases (5.08%)—follicular/glandular, one case (1.69%)—with giant cells, and one case (1.69%)—with small cells (Figure 1). Multifocality was found in nine cases (15.25%) of MTC, four of them belonging to the familial MTC category. In nine cases (15.25%), none of them were familial, and other collision tumors (PTC uni- or multifocal) were detected. Accompanying thyroid pathology included nodular goiter—28 cases (47.45%), colloid goiter—24 cases (40.67%) including all familial MTC patients, and Hashimoto thyroiditis—7 cases (11.86%).

The lymphovascular invasion was present in 34 cases (57.62%), including four cases of familial MTC. Perineural invasion was noticed in two cases (3.38%) of sporadic MTC.

An extrathyroidal extension was detected in 24 cases (40.67%), two of them being familial.

Complete tumor resection with negative margins (R0) was pathologically certified in 53 cases (89.83%), including all four familial cases. Incomplete tumor resection with positive edges (R1) was identified in six cases (10.16%).

Within the study group, cases were classified in T stages as follows: 26 cases (44.06%) in T1, eight cases (13.5%), two of them familial MTC, in T2, 24 cases (40.67%), two of them familial, in T3, and one case (1.69%) in T4a.

According to the lymph node status of the 43 cases that benefited from lymph node dissection, 23 cases (53.48%) were classified in stage N0 and 20 cases (46.52%) in stage N1 (four cases—N1a, 16 cases including two familial MTC—N1b).

Recurrence of disease was reported in 14 cases (23.7%), 11 cases (1 familial MTC) with lymph node involvement only, and three cases with lymph node and visceral metastases (one familial case located in the lung, one case in bone, and one case in cervical and bilateral ovarian sites). No morphological differences were observed between primary tumors and secondary lymph nodes or visceral metastatic determinations.

The preoperative calcitonin was available for 41 cases with a minimum value of 35 pg/mL, a maximum value of 2000 pg/mL, and a median value of 446.5 pg/mL. We found no statistically significant correlation between preoperative calcitonin and tumor size (*p* = 0,056) or lymph node metastases (*p* = 0.376).

For the 21 cases (36%) in which calcitonin was evaluated both preoperatively and postoperatively, serum calcitonin levels showed elevated preoperative values between 38–2000 pg/mL and normalized postoperative values (between 2–11.5 pg/mL) or persisted at increased levels (between 21–2000 pg/mL); in cases with familial MTC, two patients presented postoperative normalized calcitonin whereas the other two maintained high calcitonin levels. Table 2 resumes the serum calcitonin level according to pTN staging. In cases where surgical treatment provided complete tumor excision and lymph node dissection, calcitonin levels normalized and remained constant during the 3 months of patient follow-up—an excellent predictor of complete remission. Postoperative calcitonin levels remained elevated in association with lymph node metastasis and distant metastasis.

The follow-up with the patients was carried out at an interval between 5 and 7 years, the most frequent relapse (lymph node metastases) being registered at about 12 months after diagnosis. The visceral metastases were identified after 6 years. In this period of time, only one patient died.

### 3.2. Grading System Parameters

In the study group, the mitotic index was ≤ 2 in 16 cases (27.12%), between 3 and 4 in 22 cases (37.28%), and between 5 and 10 in 21 cases (35.60%). We registered a Ki67 proliferative index < 2.9% in 44 cases (74.56%), between 3 and 4.9% in 9 cases (15.26%), and between 5 and 10% in 6 cases (10.18%). Tumor necrosis was present in three cases (5.09%) and absent in 56 cases (94.91%). Of all cases, only six patients (10.17%) presented both a mitotic index and Ki67 proliferative index above the threshold value of 5, of which, two of them were associated with necrosis. Since MTC is classified as high-grade based on the presence of at least one of the three criteria, 21 cases (35.58%) met the conditions for high-grade; the other 38 (64.42%) were classified as low-grade.

**Figure 2:**
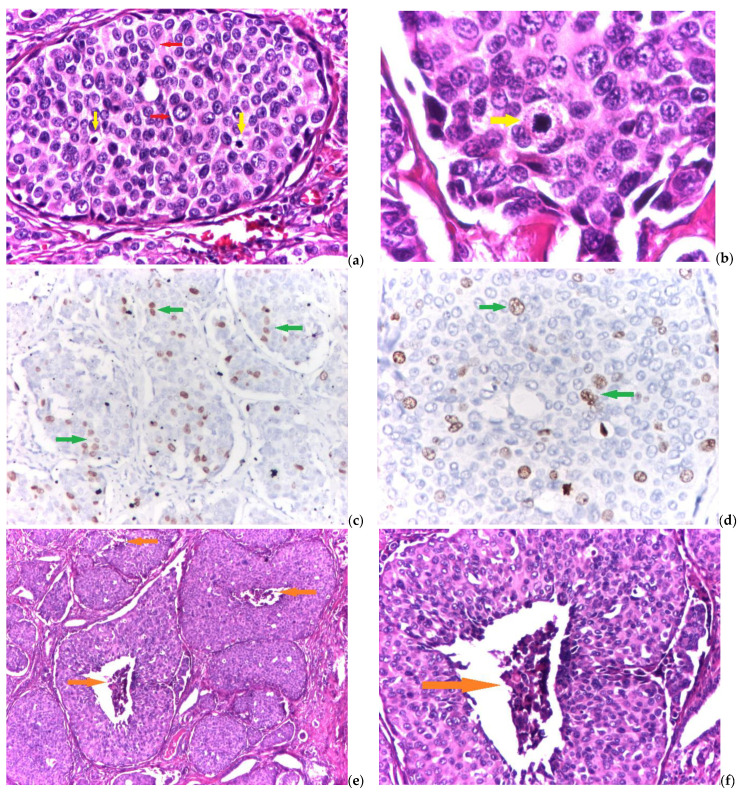
(**a**–**f**) Grading system parameters for high-grade medullary thyroid carcinoma: (**a**) solid area with nuclear pleomorphism—red arrows and two mitosis—yellow arrows (hematoxylin and eosin—H and E, magnification 10×), (**b**) detail for atypical mitosis—yellow arrow (hematoxylin and eosin—H and E, magnification 40×), (**c**) numerous Ki67 positive cells—green arrows—in solid tumor areas (immunohistochemical reaction—IHC, antiKi67 antibody, magnification 10×), (**d**) detail for Ki67 proliferative index—green arrows (immunohistochemical reaction—IHC, antiKi67 antibody, magnification 20×), (**e**) multiple foci of prominent tumor necrosis—orange arrows (hematoxylin and eosin—H and E, magnification 4×), and (**f**) detail for necrotic cells and karyorrhectic nuclear detritus—orange arrow (hematoxylin and eosin—H and E, magnification 10×).

The detailed results obtained in the assessment of the parameters used for the grading system are summarized in Table 3, while Figure 2 shows the relevant aspects of high-grade MTC.

The statistical analysis showed no significant correlations between the tumor grade (low versus high) and classical clinicopathological characteristics (gender, age, tumor size, histological variant, focality, resection margins, recurrence, and coexisting thyroid pathology).

### 3.3. Relationships between Clinicopathological Prognostic Factors

In evaluating the impact of clinicopathological factors with prognostic potential in tumor behavior, we analyzed the correlations between three parameters considered as elements of aggression—namely extrathyroidal extension, lymphovascular invasion, and lymph node metastasis—and age, tumor size, histological variant, focality, resection margins, tumor recurrence, related thyroid pathology, and the high- or low-grade of the tumor, established according to the international MTC grading system.

#### 3.3.1. Extrathyroidal Extension

Our results showed the absence of any statistically significant correlation between the extrathyroidal extension and classical clinicopathological characteristics, including the parameters of the MTC grading system, when assessed individually and integrated by reporting low-grade and high-grade risk groups. Thus, reporting to OR has no statistical relevance (Table 4).

#### 3.3.2. Lymphovascular Invasion

Statistical analysis revealed significant correlations between lymphovascular invasion (present vs. absent) and tumor size (*p* < 0.0001, OR = 13.695) (Table 5). Association between these histopathological features indicated a risk rate of 13.695 for lymphovascular invasion in tumors larger than 40 mm.

No statistically significant correlation was found for the other classical clinicopathological characteristics, including the parameters of the MTC grading system, when assessed individually and integrated by reporting low-grade and high-grade risk groups.

#### 3.3.3. Lymph Node Metastasis

There were recorded statistically significant correlations between lymph node metastasis (present vs. absent) and tumor size (*p* < 0.022, OR = 6), and tumor focality (*p* < 0.023, OR = 9.428) (Table 6). Association between these histopathological features indicated, for lymph node metastasis, a risk rate of 6 for tumors larger than 40 mm, and of 9.42 for multifocal tumors.

No statistically significant correlation was found for the other classical clinicopathological characteristics, including the parameters of the MTC grading system, when assessed individually and integrated by reporting low-grade and high-grade risk groups.

## 4. Discussion

The first series of MTC described in 1959 comprised 21 cases characterized by an intermediate grade of aggressiveness in comparison with PTC or follicular thyroid carcinoma and anaplastic thyroid carcinoma; 12 patients presented lymph node metastases at the time of diagnosis, and eight patients subsequently developed distant metastases [2].

Over the last 20 years, the incidence of MTC has been steady with a low percentage (2–3%) of MTC reported among all thyroid tumors, and the mortality has remained unchanged [1,23,24,25]. However, the major problem of MTC remains in the validation of the prognostic factors conferring worse outcomes. Investigation of some representative series of cases highlighted the fact that tumor size, extrathyroidal extension, lymph node, and distant metastases may be considered predictors of survival [1,6,10,23,26,27,28].

Starting from this framework of knowledge, the analysis of the clinicopathological profile of our series of 59 cases may contribute to the concerted effort of specialists in endocrine pathology, aiming to identify possible differences in the biological potential of aggression in MTC. It is worth emphasizing that our study also includes the classification of MTC as low-grade and high-grade using the new international MTC grading system [12].

### 4.1. Significance of Classical Clinicopathological Parameters

In our study group, we recorded a higher frequency of female cases (46 cases—80%), compared to males (13 cases—20%)—similar data to those reported in the literature [1,6,27]. The female predominance can be attributed to the hormonal substrate known as a potential tumor modulator [1,5]. Consistent with other reports [5,6,26], female patients presented a more advanced stage of the disease (pT2, pT3), older age (>55 years), and a more unfavorable clinical evolution, supported by tumor recurrence (10 cases—17%) and distance metastasis (two cases—3.3%).

The analysis of age in relation to other clinicopathological features led to additional information that may contribute to deepening knowledge of the biological profile of MTC, with correlation to personalized therapeutic protocols. In our study group with a mean age of approximately 60 yo, we found that patients over the age of 55 had a more severe course of the disease. Specifically, the patients over 55 years old represented 70% of all cases with extrathyroidal extension (17 from 24) and lymphovascular invasion (24 from 34) and 60% of all cases with lymph node metastasis (12 from 20)—with repercussions in T3 and N1b staging. Thus, our results sustain the significance of age in assessing prognosis, in line with previously published reports [5,6,27].

Tumor size is an important clinicopathological characteristic used in tumor staging, bringing information with definite prognostic value on the evolution of the disease, mainly for overall survival, the association of lymph node or distant metastasis, and tumor recurrence, respectively [1,5,21]. In our study group, the size of the tumor nodules was variable, being generally larger in the elderly. Patients over 55 yo had an average size of 40 mm, and patients under 55 yo had predominantly dimensions between 10 and 25 mm, an aspect also reported in the literature [5,6,26,27,28,29].

The circumscribed appearance, a characteristic of the tumor nodule in MTC in the initial stages of tumor development, was present in most cases in the pT1a and pT1b stages and only in a few cases in the pT2 stage. However, this feature is less applicable in the case of tumors classified in the pT3 and pT4 stages because the delimitation from the periphery is either absent or only partially present, dominating the infiltrative character.

Although the presence of lymph node metastases in tumors smaller than 10 mm that benefited from radical/modified radical lymph node dissection is reported in 10–30% of cases [1,30], we have no case of node involvement. Of the 17 cases (28.8%) with sizes between 10 and 20 mm, two cases (11.76%) were associated with lymph node metastasis, and of the 21 cases (35.5%) with sizes between 20 and 40 mm, 10 cases (47.6%) had positive lymph nodes; these values are close to those cited in the literature [1]. In the 12 cases (20.3%) larger than 40 mm, lymph node metastasis was present in eight cases (66.6%). Consequently, our results indicate a progressive growth percentage in lymph node metastasis in parallel with the increase in tumor size.

Identification of histological MTC subtypes is a constant concern in routine diagnosis, but these variants do not significantly influence prognosis [1]. No significant differences between the MTC variants and aggression parameters were found. A particular aspect identified was the coexistence of MTC with PTC (with different cell origins) in seven cases (12%). This association is rare, being reported in 4 to 13.8% of cases [5,31].

The presence of multiple tumor lesions is characteristic of familial MTC [1,5,27]. Of the nine multifocal cases (15.2%), four cases (44.4%) were classified based on clinical information as familial MTC. On this point, we should comment on the lack of confirmation for RET proto-oncogene mutations as an important limitation of our study.

At intervals of 12–48 months, we registered locoregional recurrence (stations I, II, III, IV, or V) in 14 cases (23.7%), three of them (1.7%) associating visceral metastases (one case in lung, one case in bone, and one case in cervical and bilateral ovarian location). The cases with recurrences presented, at the first surgical intervention, unilateral or bilateral cervical nodal involvement, aspects that correlate with advanced staging (pT3N1b). An important aspect analyzed in our study was the disease-free period until the onset of recurrence appeared. Patient follow-up showed that the disease-free period was significantly longer in pT1 and pT2 cases without lymph node involvement compared to pT3 cases with metastasis. The results obtained are comparable to data published in the mainstream [1,5,31].

The assessment of the resection margins is critical to the prognostic of MTC because a microscopically positive margin is generally associated with a worse course of the disease and consequently with a poor prognosis. Moreover, the status of resection margins is correlated with therapeutic management. Thus, radiotherapy is recommended only after nonradical surgery with positive margins [32]. In our series, we documented a total tumor resection with negative margins (R0) in approximately 90% of cases—a fact that creates circumstances for a favorable prognosis.

### 4.2. Significance of the Grading System

The new proposed MTC grading system aims to implement a grading tool for MTC in line with similar systems validated for other neuroendocrine tumors with origins in the neural crest [12]. By applying the mitotic count, Ki67 proliferative index, and tumor necrosis, the 59 cases in our series were stratified into two risk groups as follows: 21 cases in the high-grade group and 38 cases in the low-grade group. We found no statistically significant correlation between the tumor grade, classical clinicopathological characteristics, and parameters for tumor aggressiveness (extrathyroidal extension, lymphovascular invasion, and lymph node metastasis). This is worth mentioning that the MTC grading system was proposed in November 2021, so results obtained after its application are still unreported. Therefore, we were able to compare our data only with the data in Xu’s article [12], and the results were inconsistent. The work done by Xu et al. demonstrated statistically significant differences among MTC of different histologic grades and gender, tumor size, postoperative CEA, distant metastasis at presentation, stage group, lymphovascular invasion, resection margin, serum calcitonin doubling time, and external beam radiotherapy [12]. Despite these inconsistencies, we consider that the criteria included in the new proposed MTC grading system are excellent markers for the identification of the risk of aggressive behavior. In our opinion, the contradictory results should be explained by the relatively small number of cases in our series—an element that is an important limitation of our study.

### 4.3. Significance of Aggressiveness Parameters

In the 2004 WHO classification, extrathyroidal extension in perithyroidal adipose tissue was introduced as a staging criterion [33]. An important change brought by the 2017 WHO classification consisted of redefining the extrathyroidal extension considering infiltration in the perithyroidal muscle tissue and not in the perithyroidal adipose tissue [1].

The analysis of classical clinicopathological factors in relation to the extrathyroidal extension did not show statistically significant differences. However, out of the total 24 cases (40.6%) of MTC with extrathyroidal extension, seven cases (29.1%) were associated with tumor recurrence, and two cases (8.3%) developed distant metastasis. These results support extrathyroidal muscle invasion as an element of aggression for tumor progression in accordance with several studies and practice guidelines that impose its assessment and report [5,27,34,35].

Of the total 59 cases of MTC, 34 (over 50%) were associated with lymphovascular invasion. A particular aspect should be highlighted: In the pT1 stage, the vascular tumor emboli did not constitute a ubiquitous element, but we found a frequent occurrence on the edge of the tumor infiltrative area in the nontumor thyroid tissue or perithyroidal adipose tissue. Special attention should be paid because tumor embolism is, by itself, a factor that facilitates tumor progression and metastasis [5,36]. Statistical analysis revealed correlations with tumor size. Moreover, our data support an OR of 13.695 for the presence of tumor emboli in tumors larger than 40 mm.

Our results reflect the dynamic relationship between tumor size, lymphovascular invasion, and tumor lymph node metastases. From the total of 43 cases for which lymph node dissection was performed, 20 cases (34%) presented nodal metastases; the frequency was lower compared to other reports (46.2%, and 55%) [5,29]. Statistical analysis showed correlations with tumor size and multifocality; the OR for nodal involvement was 6 in tumors larger than 40 mm and 9.428 in multifocal cases.

## 5. Conclusions

Our data indicate that lymphovascular invasion and lymph node metastasis remain significant markers for MTC aggressiveness. Although we did not confirm statistically significant correlations, we consider that the MTC grading system provides essential criteria for including MTC in low-grade and high-grade risk groups and optimizing the prognosis assessment from the moment of histopathological diagnosis. Thus, studies on larger series of cases are mandatory to detect and validate new factors responsible for the variable course of MTC.

## Figures and Tables

**Table 1:** The antibodies used for MTC IHC staining.

Antibody	Manufacturer	Clone	Antigen Retrieval	Class	Dilution	Labeling	Cellular Localization
AntiCgA	Leica Biosystems, Deer Park, IL, USA	5H7	Citrate, pH 6	Monoclonal mouse antihuman chromogranin A	1:400	Neuroendocrine cells	Cytoplasm
AntiSyn	Leica Biosystems, Deer Park, IL, USA	27G12	Citrate, pH 6	Monoclonal mouse antisynaptophisin antibody	1:50	Neuroendocrine cells	Cytoplasm
Anticalcitonin	Leica Biosystems, Deer Park, IL, USA	CL 1948	Citrate, pH 6	Monoclonal mouse anticalcitonin antibody	1:250	C cells	Cytoplasm
AntiCD56	Agilent Dako, Santa Clara, CA, USA	123C3	Citrate, pH 6	Monoclonal mouse antiCD56 antibody	1:75	Neuroendocrine cells	Membrane
AntiTTF1	Agilent Dako, Santa Clara, CA, USA	mAb 8G7G3/1	pH 9	Monoclonal mouse antiTTF1 antibody	1:50	Follicular thyroid cells	Nuclear
AntiKi67	ThermoFisher Scientific, Waltham, MA, USA	SP6	Citrate, pH 6	Monoclonal rabbit antiKi67 antibody	1:250	Proliferating cells	Nuclear

**Table 2:** Pre- and postoperative calcitonin value corresponding to pTN staging.

Preoperative Calcitonin Value				Postoperative Calcitonin Value			
pTN	Order of Tens (n = 7)	Order of Hundreds (n = 10)	Order of Thousands (n = 4)	Normal Values (≤11.5 pg/mL) (n = 12)	Order of Tens (n = 4)	Order of Hundreds (n = 4)	Order of Thousands (n = 1)
T1	3 (14.2%)	5 (23.8%)	1(4.7%)	8 (38.0%)	1 (4.7%)	0 (0%)	0 (0%)
T2	3 (14.2%)	3 (14.2%)	0 (0%)	3 (14.2%)	2 (9.5%)	1 (4.7%)	0 (0%)
T3	1 (4.7%)	2 (9.5%)	3 (14.2%)	1 (4.7%)	1 (4.7%)	3 (14.2%)	1 (4.7%)
N1	0 (0%)	3 (14.2%)	2 (9.5%)	0 (0%)	3 (14.2%)	1 (4.7%)	1 (4.7%)
N0	3 (14.2%)	5 (23.8%)	0 (0%)	9 (42.8%)	0 (0%)	1 (4.7%)	0 (0%)
Nx	4 (19.0%)	2 (9.5%)	2 (9.5%)	3 (14.2%)	1 (4.7%)	2 (9.5%)	0 (0%)

n—number of cases.

**Table 3:** Classification in low-grade and high-grade classes according to nternational Medullary Thyroid Carcinoma Grading System.

Grading System Parameter	Total MTC Cases (n = 59)	
	Low-Grade MTC n, %	High-Grade MTC n, %
Mitotic index		
0	0 (0%)	
1	8 (13.56%)	
2	8 (13.56%)	
3	10 (16.95%)	
4	12 (20.33%)	
5		11 (18.64%)
6		1 (1.70%)
7		8 * (13.56%)
8		0 (0%)
9		0 (0%)
10		1 # (1.70%)
Ki67 proliferative index		
0–1.9%	22 (37.28%)	
2–2.9%	22 (37.28%)	
3–3.9%	7 (11.86%)	
4–4.9%	2 (3.40)	
5–5.9%		1 ** (1.70%)
6–6.9%		3 ** (5.08%)
7–7.9%		0
8–8.9%		1 ^ (1.70%)
9–10%		1 ** (1.70%)
Tumor necrosis		
Absent	56 (94.91%)	
Present		3 (5.09%)

MI—mitotic index, *—5 cases with Ki67 > 5, #—1 case with Ki67 > 5, **—1 case with IM 7, ^—1 case with IM 10.

**Table 4:** Clinicopathological characteristics of MTC in relation to extrathyroidal extension.

Clinicopathological Characteristics	Extrathyroidal Extension		Univariate Analysis	OR (95% CI)
	Present (n = 24)	Absent (n = 35)		
Age at diagnosis				
<55 yo	7 (29.1%)	10 (28.5%)	0.964	0.971 (0.308–3.054)
>55 yo	17 (70.8%)	25 (71.4%)		
Tumor size (mm)				
<10 mm	1 (4.1%)	8 (22.8%)	0.088	1.925 (0.516–7.177)
10–40 mm	16 (66.6%)	22 (62.8%)		
>40 mm	7 (29.1%)	5 (14.2%)		
Histological variant				
Conventional	12 (50%)	23 (65.7%)	0.227	1.916 (0.662–5.542)
Other variants	12 (50%)	12 (43.8%)		
Focality of the tumor				
Unifocal	18 (75%)	32 (91.4%)	0.084	3.555 (0.792–15.957)
Multifocal	6 (25%)	3 (8.5%)		
Resection margins				
R0	20 (83.33%)	33 (94.29%)	0.190	3.300 (0.553–19.685)
R1	4 (16.67%)	2 (5.71%)		
Tumor recurrence				
present	7 (29.1%)	7 (20%)	0.418	1.647 (0.492–5.516)
absent	17 (70.9%)	28 (80%)		
Coexisting thyroid pathology				
Colloid goiter †	11 (45.8%)	13 (37.1%)	0.700	0.545 (0.096–3.075)
Nodular goiter	11 (45.8%)	17 (48.5%)		
Hashimoto thyroiditis	2 (8.3%)	5 (14.2%)		
Mitotic index				
<5/2 mm^2^ (low-grade)	14 (58.33%)	24 (68.57%)	0.421	1.558 (0.529–4.592)
>5/2 mm^2^ (high-grade)	10 (41.67%)	11 (31.43%)		
Ki67 proliferative index				
<5% (low-grade)	20 (83.33%)	33 (94.29%)	0.190	3.300 (0.553–19.685)
>5% (high grade)	4 (16.67%)	2 (5.71%)		
Tumor necrosis				
absent	23 (95.83%)	33 (94.29%)	0.790	0.717 (0.061–8.387)
present	1 (4.17%)	2 (5.71%)		
Tumor grade				
high-grade	10 (41.67%)	11 (31.43%)	0.421	1.558 (0.529–4.592)
low-grade	14 (58.33%)	24 (68.57%)		

n: number of cases; OR: odd ratio; CI: confidence interval; † colloid and nodular goiter vs. Hashimoto thyroiditis.

**Table 5:** Clinicopathological characteristics of MTC in relation to lymphovascular invasion.

Clinicopathological Characteristics	Lympho-Vascular Invasion		Univariate Analysis	OR (95% CI)
	Present (n = 34)	Absent (n = 25)		
Age at diagnosis				
<55 yo	10 (29.4%)	7 (28%)	0.905	0.933 (0.297–2.927)
>55 yo	24 (70.5%)	18 (72%)		
Tumor size (mm)				
<10 mm	0 (0%)	9 (36%)	0.0001 *	13.695 (1.662–112.846)
10–40 mm	23 (67.6%)	15 (60%)		
>40 mm	11 (32.4%)	1 (4%)		
Histological variant				
Conventional	19 (55.8%)	16 (64%)	0.398	0.664 (0.257–1.717)
Other variants	15 (44.1%)	19 (76%)		
Focality of the tumor				
Unifocal	27 (79.4%)	23 (92%)	0.183	2.981 (0.562–15.790)
Multifocal	7 (20.5%)	2 (8%)		
Resection margins				
R0	29 (85.3%)	24 (96%)	0.179	4.138 (0.452–37.875)
R1	5 (14.7%)	1 (4%)		
Tumor recurrence				
Present	10 (29.4%)	4 (16%)	0.238	2.187 (0.597–8.019)
Absent	24 (70.6%)	21 (84%)		
Coexisting thyroid pathology				
Colloid goiter †	14 (41.1%)	10 (40%)	0.2307	0.250 (0.0442–1.4136)
Nodular goiter	18 (52.9%)	10 (40%)		
Hashimoto thyroiditis	2 (5.8%)	5 (20%)		
Mitotic index				
<5/2 mm^2^ (low-grade)	19 (55.9%)	19 (76%)	0.111	2.500 (0.799–7.821)
>5/2 mm^2^ (high-grade)	15 (44.1%)	6 (24%)		
Ki67 proliferative index				
<5% (low-grade)	29 (85.3%)	24 (96%)	0.179	4.138 (0.452–37.875)
>5% (high grade)	5 (14.7%)	1 (4%)		
Tumor necrosis				
absent	31 (91.2%)	25 (100%)	0.127	0.554 (0.438–0.700)
present	3 (8.8%)	0 (0%)		
Tumor grade				
high-grade	15 (44.1%)	6 (24%)	0.111	2.500 (0.799–7.821)
low-grade	19 (55.9%)	19 (76%)		

n: number of cases; OR: odd ratio; CI: confidence interval; * *p*-value < 0.05 was considered to be statistically significant; † colloid and nodular goiter vs. Hashimoto thyroiditis.

**Table 6:** Clinicopathological characteristics of MTC according to lymph node metastasis.

Clinicopathological Characteristics	Lymph Node Metastasis		Univariate Analysis	OR (95% CI)
	Present (n = 20)	Absent (n = 23)		
Age at diagnosis				
<55 yo	8 (40%)	5 (21.7%)	0.193	0.416 (0.109–1.583)
>55 yo	12 (60%)	18 (78.2%)		
Tumor size (mm)				
<10 mm	0 (0%)	3 (13%)	0.022 *	6 (1.081–33.275)
10–40 mm	12 (60%)	18 (78.2%)		
>40 mm	8 (40%)	2 (8.6%)		
Histopathologic MTC type				
Conventional	12 (60%)	12 (52,1%)	0.606	0.727 (0.216–2.444)
Other variants	8 (40%)	11 (47.8%)		
Focality of the tumor				
Unifocal	14 (70%)	22 (95.6%)	0.023 *	9.428 (1.023–86.860)
Multifocal	6 (30%)	1 (4.4%)		
Resection margins				
R0	16 (80%)	22 (95.6%)	0.110	5.500 (0.560–53.986)
R1	4 (20%)	1 (4.3%)		
Tumor recurrence				
present	6 (30%)	5 (21.7%)	0.536	1.543 (0.389–6.115)
absent	14 (70%)	18 (78.3%)		
Coexisting thyroid pathology				
Colloid goiter †	8 (40%)	10 (43.4%)	0.967	1.125 (0.308–4.104)
Nodular goiter	9 (45%)	10 (43.4%)		
Hashimoto thyroiditis	3 (15%)	3 (13%)		
Mitotic index				
<5/2 mm^2^ (low-grade)	11 (55%)	16 (69.6%)	0.324	1.870 (0.535–7.776)
>5/2 mm^2^ (high-grade)	9 (45%)	7 (30.4%)		
Ki67 proliferative index				
<5% (low-grade)	16 (80%)	22 (95.6%)	0.110	5.500 (0.560–53.986)
>5% (high grade)	4 (20%)	1 (4.4%)		
Tumor necrosis				
Absent	19 (95%)	22 (95.6%)	0.919	1.158 (0.068–19.798)
Present	1 (5%)	1 (4.4%)		
Tumor grade				
High-grade	9 (45%)	7 (30.4%)	0.324	1.870 (0.535–6.534)
Low-grade	11 (55%)	16 (69.6%)		

n: number of cases; OR: odd ratio; CI: confidence interval; * *p*-value < 0.05 was considered to be statistically significant; † colloid and nodular goiter vs. Hashimoto thyroiditis.

## Data Availability

The data used to support the findings of this research are available upon request to the authors.
